# Efficacy of rebamipide for the treatment of dry eye disease: An updated meta-analysis of randomized and non-randomized controlled trials

**DOI:** 10.1097/MD.0000000000048424

**Published:** 2026-05-01

**Authors:** Meer Murtaza, Umar Farooque, Asia Batool, Maria Rasool, Nazeer Ahmed, Syeda Hafsa Qadri, Makhzan Ali Akbar, Shahroz Ali, Tirath Patel

**Affiliations:** a Department of Internal Medicine, Liaquat University of Medical and Health Sciences, Jamshoro, Pakistan; b Department of Internal Medicine, Health Education England, East of England, United Kingdom; c Department of Internal Medicine, Sir Syed College of Medical Sciences, Karachi, Pakistan; d Department of Internal Medicine, King Edward Medical University, Lahore, Pakistan; e Department of Internal Medicine, Karachi Medical and Dental College, Karachi, Pakistan; f Department of Internal Medicine, Dow University of Health Sciences, Karachi, Pakistan; g Department of Internal Medicine, North Sichuan Medical College, Nanchong, China; h Department of Internal Medicine, Karachi Metropolitan University, Karachi, Pakistan; i Department of Internal Medicine, Trinity Medical Sciences University School of Medicine, Kingstown, Saint Vincent and the Grenadines.

**Keywords:** dry eye disease, meta-analysis, ophthalmic suspension, optical quality, rebamipide 2%, safety, systematic review

## Abstract

**Background:**

Dry eye disease (DED) is a multifactorial ocular surface disorder characterized by tear film instability and inflammation. Rebamipide 2% ophthalmic suspension, a mucin secretagogue, has been investigated as a potential treatment due to its unique mechanism targeting mucin deficiency and ocular surface repair.

**Methods:**

A systematic search of PubMed, Embase, and Cochrane Library was conducted for studies published between January 2013 and March 2025. Non-randomized and randomized controlled trials evaluating topical 2% rebamipide in patients with DED were included. Outcomes assessed included tear breakup time, Schirmer *I* test, fluorescein staining scores, ocular surface disease index, and adverse events. Data were synthesized using standard meta-analytic techniques and subgroup analyses.

**Results:**

Thirteen studies, including both randomized and non-randomized trials, were analyzed, comprising a total of 575 participants. Meta-analysis showed that rebamipide nonsignificantly increased tear breakup time at 2 weeks (standardized mean difference [SMD] = 1.04; 95% confidence interval (CI):–0.94 to 3.03; *P* = .30; *I*^2^ = 89.3%), significant at 4 weeks (SMD = 1.19; 95% CI: 0.74–1.64; *P* < .0001; *I*^2^ = 85%) and nonsignificant at 12 weeks (SMD = 0.97; 95% CI:–0.15 to 2.08; *P* = .09; *I*^2^ = 80.1%) indicating enhanced tear film stability. Schirmer *I* test values showed no significant improvement (SMD = 0.04; 95% CI: −0.35–0.43; *P* = .83), suggesting limited effect on aqueous tear production. Fluorescein staining scores showed a reduction approaching statistical significance (SMD = −0.68; *P* = .051), while symptom scores measured by ocular surface disease index trended toward improvement, also approaching significance (SMD = −1.17; *P* = .055). Subgroup analyses revealed greater efficacy in contact lens wearers and postsurgical patients. Safety analysis indicated excellent tolerability, with a high adherence rate (96.8%) and only mild adverse effects such as dysgeusia and nasopharyngitis.

**Conclusion:**

Rebamipide 2% ophthalmic suspension improves tear stability and ocular surface health in mucin-deficient DED with a favorable safety profile. Further high-quality trials in diverse populations are warranted to confirm its role in global clinical practice.

## 1. Introduction

Dry eye disease (DED) is a prevalent and multifactorial ocular surface disorder, characterized by tear film instability, hyperosmolarity, ocular surface inflammation, and mucin-layer dysfunction. These interconnected pathophysiological mechanisms lead to symptoms such as ocular discomfort, visual disturbance, and potential epithelial damage, ultimately contributing to a self-perpetuating cycle of irritation and ocular surface breakdown.^[1,2]^ As a result, DED not only impairs visual function but also significantly reduces quality of life, imposing a considerable psychological and economic burden on affected individuals and healthcare systems alike.^[3,4]^

From a global perspective, DED affects approximately 5% to 50% of the adult population, with its prevalence varying based on diagnostic criteria, age distribution, and environmental exposures.^[5]^ For instance, older adults frequently suffer from DED due to age-related tear dysfunction and systemic comorbidities, with prevalence rates reported between 22% to 34% in individuals over the age of 60.^[6]^ Conversely, younger populations, particularly university students, have shown prevalence rates as high as 68%, primarily associated with prolonged digital screen use and reduced blink frequency.^[7]^ In addition to age and behavior, several risk factors have been identified, including low humidity, air pollution, autoimmune diseases, diabetes mellitus, and the use of systemic or topical medications.^[5]^

The underlying pathophysiology of DED has been extensively documented in both foundational and recent literature. Seminal contributions, such as the National Eye Institute/Industry Workshop report by Lemp (1995), provided the initial diagnostic and classification framework for DED.^[8]^ These early efforts were further refined by the TFOS DEWS I (2007) and DEWS II (2017) reports, which emphasized the central role of inflammation, tear film instability, and mucin deficiency in disease progression.^[9,10]^ Despite this growing understanding, conventional treatments such as artificial tears, anti-inflammatory agents (e.g., cyclosporine 0.05% and lifitegrast 5%), and lubricating gels primarily offer symptomatic relief and often fail to effectively target the underlying inflammatory processes or restore long-term tear film stability.^[11]^

In this context, rebamipide 2% ophthalmic suspension, originally developed as a gastrointestinal mucosal protectant, has emerged as a novel therapeutic candidate for DED management due to its mucin secretagogue properties. Mechanistically, rebamipide promotes goblet cell proliferation and enhances the expression of mucin genes, including MUC1, MUC4, MUC5AC, and MUC16. This action stabilizes the tear film and reduces ocular surface inflammation through epidermal growth factor receptor-mediated pathways.^[12–15]^ Furthermore, the agent increases levels of prostaglandin E2 and I2 while scavenging free radicals, thereby providing additional protection to the ocular surface.^[14]^ Rebamipide has been approved for ophthalmic use in several Asian countries and has demonstrated promising efficacy across diverse patient populations and clinical scenarios.^[16–18]^

Supporting evidence from multiple clinical studies has shown that rebamipide treatment is associated with improvements in tear film breakup time (TBUT), corneal and conjunctival staining scores, Schirmer test values, and subjective symptom relief. Moreover, therapeutic benefits have been observed in specific subgroups such as contact lens wearers and patients undergoing cataract surgery.^[19–22]^ However, notable heterogeneity in study design, participant demographics, and outcome assessment tools has limited the generalizability and integration of these findings into standardized clinical practice.

Therefore, to provide a comprehensive synthesis of the available evidence, we conducted a systematic review and meta-analysis of controlled clinical trials published between 2013 and 2025. Our objective was to evaluate the efficacy, safety, and ocular surface outcomes associated with rebamipide 2% ophthalmic suspension in the treatment of DED. In addition to assessing therapeutic outcomes, we critically examined the methodological quality of included studies and explored rebamipide’s potential role in modulating conjunctival goblet cell density, an important indicator of ocular surface integrity and function.

## 2. Methods and materials

### 2.1. Search strategy and databases

This meta-analysis was performed according to the Preferred Reporting Items for Systematic Reviews and Meta-Analyses (PRISMA)^[23]^ and registered in PROSPERO (CRD42023474427). A comprehensive literature search was conducted across various electronic databases, including PubMed, Embase, and Cochrane, to identify relevant studies published between January 1, 2013, and March 2025. The search aimed to retrieve randomized clinical trials (RCTs) and non-RCTs evaluating the efficacy of ophthalmic treatments for DED. In PubMed, the following search strategy was used:(“dry eye” OR “dry eye syndrome” OR “DED” OR “evaporative dry eye” OR “keratoconjunctivitis sicca” OR “ocular surface disease”) AND (“rebamipide ophthalmic suspension” OR RBM OR “OPC 12759” OR “OPC-12759” OR “rebamipide”) AND (“ocular surface disease index (OSDI)” OR OSDI OR “Schirmer *1* test” OR “tear breakup time” OR TBUT OR “fluorescein corneal staining score” OR FCS).

### 2.2. Inclusion criteria

The inclusion criteria were that studies included patients with dry eye who were treated with topical 2% rebamipide ophthalmic solution administered 4 times daily. Signs and symptoms were analyzed before and at least 2 weeks after rebamipide administration. Inclusion criteria for dry eye included Schirmer *I* test ≤ 5 mm (tear-deficient type) or TBUT ≤ 5 seconds (short BUT type) with dry eye-related subjective symptoms. All the studies included were conducted following the scientific principles of the World Medical Association Declaration of Helsinki and were approved by the respective regional clinical research ethics committee. This systematic review and meta-analysis are based solely on previously published data and did not require ethical approval or informed consent.

### 2.3. Exclusion criteria

Studies were excluded if they met any of the following: publication type was a review, meta-analysis, correspondence, case report, correction, abstract, or conference proceeding; conducted on animals; used non-topical rebamipide formulations; lacked sufficient data on clinical outcomes; or included patients with conditions that could confound results, such as diffuse lamellar keratitis, ocular infections, interface opacity, trauma, nasolacrimal abnormalities, punctal occlusion (permanent or temporary), meibomian gland dysfunction, or Sjögren’s syndrome: unless these subgroups were analyzed separately.

### 2.4. Outcomes

This review assessed both efficacy and safety outcomes of 2% rebamipide ophthalmic solution in dry eye disease. Primary efficacy outcomes included TBUT, Schirmer *I* test, fluorescein staining scores (FSS) (ocular, corneal, conjunctival), and OSDI. Subgroup analyses were performed based on follow-up duration, study design (RCT vs non-RCT), and patient characteristics. Secondary outcomes included treatment compliance and safety. Methodological assessments included sensitivity analyses, publication bias evaluation, and risk of bias.

### 2.5. Data extraction and synthesis

Two independent reviewers extracted key data from each study, including author, publication year, study design, sample size, treatment duration, mean age, sex distribution, number of eyes analyzed, inclusion criteria, treatment details for rebamipide and control groups, dosage regimen, and conflicts of interest. Outcomes included TBUT (seconds), Schirmer test without anesthesia (mm),^[24]^ total corneal fluorescein staining using the NEI (0–15) or van Bijsterveld (0–9) scales, OSDI, adverse events, compliance, and authors’ overall conclusions.

Baseline and final values were collected for each outcome. Intragroup outcomes were calculated as the change from baseline to final visit, and inter-group differences were computed as the net difference between groups. Mean ± SD was used for continuous variables. Data synthesis followed Cochrane’s Synthesis Without Meta-analysis guidelines.^[25]^

### 2.6. Quality assessment

Study quality was assessed using the Cochrane Risk of Bias (RoB) 2.0 tool^[26]^ for RCTs (Fig S1, Supplemental Digital Content, https://links.lww.com/MD/R718), the National Institutes of Health Quality Assessment Tool for Before-After (Pre-Post) Studies With No Control Group for non-RCTs^[27]^ (Table S1, Supplemental Digital Content, https://links.lww.com/MD/R717), and Joanna Briggs Institute Critical Appraisal Checklist for Case Series^[28]^ (Table S2, Supplemental Digital Content, https://links.lww.com/MD/R717).

### 2.7. Statistical analysis

Statistical analysis was conducted using R version 4.4.2. Effect sizes were presented as standardized (SMD) with 95% confidence intervals (CIs). Heterogeneity was assessed using the *I*^2^ statistic. Subgroup analyses were performed for RCTs versus Non-RCTs. Publication bias was evaluated using Egger’s test and funnel plots.

## 3. Results

### 3.1. Study selection

**Figure 1.**
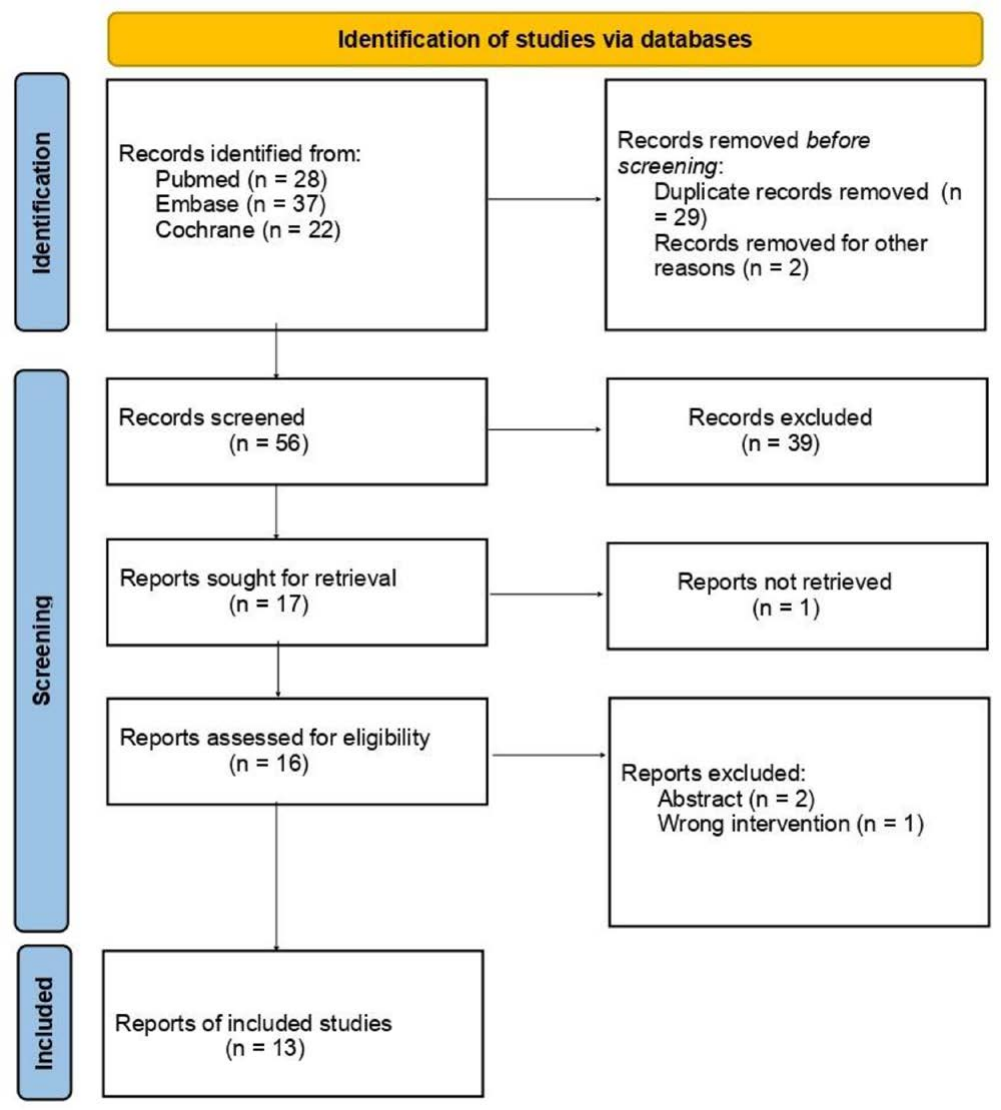
PRISMA flow diagram outlining the study selection process, from initial database search to final inclusion, in accordance with PRISMA guidelines.

A comprehensive search across PubMed, Embase, and Cochrane Library identified a total of 87 records (PubMed: n = 28, Embase: n = 37, Cochrane: n = 22). After removing 29 duplicate records and excluding 2 records for other reasons, a total of 56 records were screened for eligibility. Following title and abstract screening, 17 reports were sought for full-text retrieval. Of these, 16 reports were assessed for eligibility. Ultimately, 13 reports met the inclusion criteria and were included in the final analysis. A detailed breakdown of the study selection process is shown in the PRISMA flow diagram (Fig. 1).

### 3.2. Study characteristics

A summary of the selected unique studies is provided in Table 1. The studies were published between 2013 and 2025. A total of 13 studies^[29–41]^ were included in this meta-analysis, comprising both RCTs and non-randomized controlled trials (non-RCTs) evaluating the efficacy and safety of 2% rebamipide in DED. Among these, 10 studies were conducted in Japan, 2 in Korea, and 1 in South Korea. The included studies encompassed various subtypes of DED, such as postoperative dry eye, contact lens-associated dry eye, and video display terminal-associated dry eye, reflecting a broad clinical context.

**Table 1:** Summary of key findings from studies.

First author	Y	Study design	Country	Follow-up period (weeks)	Outcomes (methods)	N (eyes)	Sex (F/M)	Age (mean ± SD)	Dry eye status before treatment
S. Koh^[29]^	2013	Non-RCT	Japan	2, 4	TBUT, Schirmer *I* test, FSS (ocular)	26	15/1	68.5 ± 11.9	Nothing
K. Ueda^[30]^	2015	Non-RCT	Japan	2, 4, 8, 12	TBUT, Schirmer *I* test, FSS (ocular, corneal, conjunctival)	48	24/0	66.2	Nothing
T. Igarashi^[31]^	2015	Non-RCT	Japan	4	TBUT, Schirmer *I* test, FSS (ocular)	50	19/6	62.0 ± 16.6	Nothing
A. Igarashi^[32]^	2015	RCT	Japan	2, 4, 12	TBUT, Schirmer *I* test, FSS (ocular)	30	13/2	34.4 ± 10.8	After corneal refractive surgery
H. Kobashi^[33]^	2017	RCT	Japan	2, 4	TBUT, FSS (corneal)	20	09/11	72.4 ± 13.7	After PK
T. Igarashi^[34]^	2018	Non-RCT	Japan	4	TBUT, Schirmer *I* test, FSS (ocular, corneal)	40	20/0	30.0 ± 8.33	SCL wearer
T. Teshigawara^[35]^	2021	RCT	Japan	4, 12	TBUT, HOAs	30	43/15	66.57 ± 12.36	Undergo cataract surgery
T. Teshigawara^[36]^	2022	Non-RCT	Japan	4, 12	TBUT, HOAs	36	19/17	73.6 ± 3.4	After cataract surgery
Y. Sakane^[37]^	2019	Non-RCT	Japan	4, 13, 26, 52, 104	TBUT, FSS (ocular)	6	40/3	64 ± 14	Nothing
T. Teshigawara^[38]^	2022	Non-RCT	Japan	4	TBUT, HOAs	35	17/18	74.5 ± 6.2	Undergo cataract surgery
Y. Eom^[39]^	2023	RCT	Korea	4, 8, 12	TBUT, Schirmer *I* test, FSS (corneal)	72	120/26	43.3 ± 13.6	Nothing
Y. Jin et al^[40]^	2024	RCT	South Korea	4	TBUT, Schirmer *I* test, FSS (corneal, conjunctival)	15	20/8	63.1 ± 9.4	Nothing
Y. Lee^[41]^	2024	RCT	Korea	4	TBUT, Schirmer *I* test, FSS (corneal, conjunctival)	30	18/10	37.7 ± 9.7	Nothing

FSS = fluorescein staining score, HOAs = higher-order aberrations, PK = penetrating keratoplasty, RCT = randomized controlled trial, SCL = soft contact lens, TBUT = tear breakup time.

### 3.3. Efficacy outcomes

#### 3.3.1. Tear breakup time (TBUT)

A total of 13 studies were included in the meta-analysis of TBUT outcomes. At 2 weeks, 3 studies (N = 94) reported a pooled SMD of 1.04 (95% CI:–0.94 to 3.03; *P* = .30; *I*^2^ = 89.3%). This substantial heterogeneity may reflect variability in baseline dry eye severity, treatment protocols, and small sample sizes across the studies.

At 4 weeks, 12 studies (N = 366) showed a pooled SMD of 1.19 (95% CI: 0.74–1.64; *P* < .0001; *I*^2^ = 85%). The high heterogeneity observed at this time point is likely driven by differences in study design (RCTs vs non-RCTs), follow-up durations, and outcome measurement methods.

At 12 weeks, 4 studies (N = 159) demonstrated an SMD of 0.97 (95% CI:–0.15 to 2.08; *P* = .09; *I*^2^ = 80.1%). This level of heterogeneity suggests variation in long-term treatment response and patient population characteristics. Subgroup differences by follow-up duration were not statistically significant (*Q* = 0.33; *P* = .85).

Subgroup analysis by study design at 4 weeks included 5 RCTs and 7 non-randomized studies. RCTs (N = 125) showed a pooled SMD of 1.35 (95% CI: 0.67–2.03; *I*^2^ = 60.2%), while non-RCTs (N = 241) had a pooled SMD of 1.09 (95% CI: 0.34–1.85; *I*^2^ = 88.9%). The substantially higher heterogeneity among non-RCTs likely reflects inconsistencies in study protocols, population selection, and outcome assessment tools. The difference between RCTs and non-RCTs was not statistically significant (*Q* = 0.43; *P* = .51).

**Figure 2.**
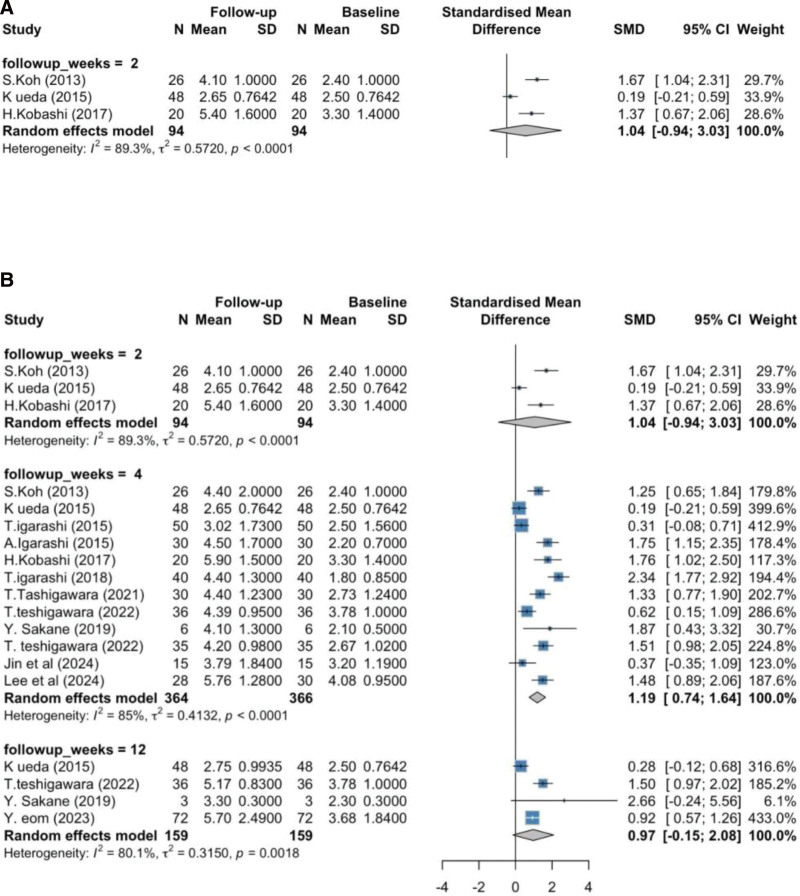
(A) Forest plot showing SMD for TBUT changes before and after rebamipide treatment at 2-week, 4-week, and 12-week follow-ups. (B) Forest plot showing subgroup analysis SMD for TBUT changes at 4 weeks between RCTs Versus Non-RCTs. CI = confidence interval, FSS = fluorescein staining scores, OSDI = ocular surface disease index, RCT = randomized clinical trial, SD = standard deviation, SMD = standardized mean difference, TBUT = tear breakup time.

The forest plots (Figs. 2A–2B) display the effect sizes and CIs for individual studies across these analyses. The largest effect sizes at 4 weeks were observed in Teshigawara (2022) (SMD 2.34, 95% CI: 1.77–2.92) and Kobashi (2017) (SMD 1.76, 95% CI: 1.02–2.50).

#### 3.3.2. Schirmer I test

Seven studies evaluated the effect of rebamipide on Schirmer *I* test values. Meta-analysis of 5 studies with a 4-week follow-up found no statistically significant improvement (SMD = 0.04, 95% CI = [−0.35, 0.43], *P* = .83; *I*^2^ = 64.1%). This moderate heterogeneity, which was higher among non-RCTs (*I*^2^ = 72.1%) compared to RCTs (*I*^2^ = 0.0%), may result from methodological differences and diverse population characteristics.

**Figure 3.**
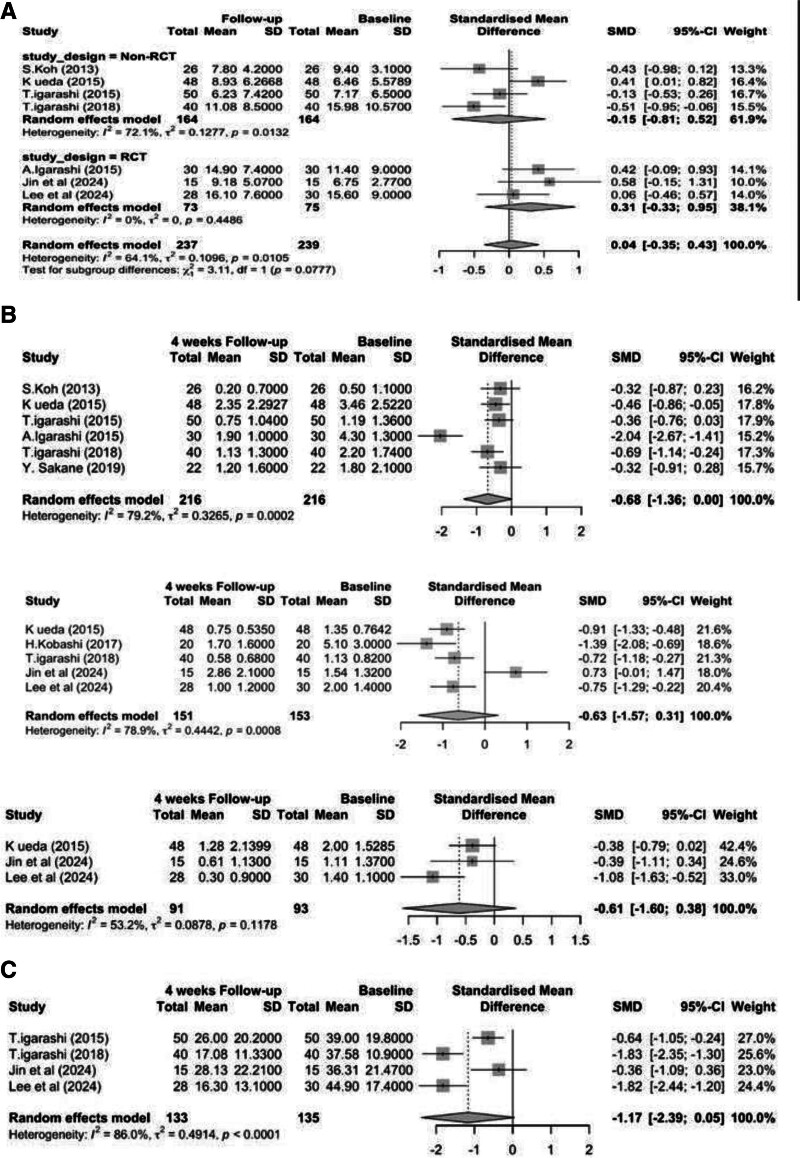
(A) Forest plot showing SMD for Schirmer *I* test changes before and after rebamipide treatment at 4-week follow-up. (B) i: Forest plot showing SMD for ocular FSS changes before and after rebamipide treatment at 4-week follow-up. (B) ii: Forest plot showing SMD for corneal FSS changes before and after rebamipide treatment at 4-week follow-up. (B) iii: Forest plot showing SMD conjunctival FSS changes before and after rebamipide treatment at 4-week follow-up. (C) Forest plot showing SMD for OSDI changes before and after rebamipide treatment at 4-week follow-up. CI = confidence interval, FSS = fluorescein staining scores, OSDI = ocular surface disease index, SD = standard deviation, SMD = standardized mean difference.

Subgroup analysis showed divergent trends between non-RCTs (SMD = −0.15) and RCTs (SMD = 0.31), but the between-group difference was not statistically significant (*P* = .078), as shown in Figure 3(A). One additional study assessed outcomes at 12 weeks, but the limited number of studies precluded meta-analysis for that time point.

#### 3.3.3. Fluorescein staining score (FSS)

Analysis of studies measuring ocular FSS at 4 weeks (*k* = 6) showed a borderline significant improvement (SMD = −0.68, 95% CI = [−1.36, 0.00], *P* = .051; *I*^2^ = 79.2%; Figure 3(B). This high heterogeneity likely reflects differences in grading systems (NEI vs van Bijsterveld), examiner interpretation, and baseline ocular surface conditions.

Corneal FSS (*k* = 5) also trended toward improvement (SMD = −0.63, 95% CI = [−1.57, 0.31], *P* = .14; *I*^2^ = 78.9%; Figure 3(B) while conjunctival FSS (*k* = 3) showed a similar trend (SMD = −0.61, 95% CI = [−1.60, 0.38], *P* = .12; *I*^2^ = 53.2%; Figure 3(B), although none reached statistical significance.

#### 3.3.4. Ocular surface disease index (OSDI)

Four studies assessing the impact of rebamipide on OSDI scores at 4 weeks reported a large but borderline significant reduction in symptoms (SMD = −1.17, 95% CI = [−2.39, 0.05], *P* = .055; *I*^2^ = 86.0%), indicating a potentially meaningful improvement in patient-reported outcomes. The substantial heterogeneity here may be attributed to variability in OSDI administration, subjective interpretation of symptom burden, and demographic differences between study populations. See Figure 3(C).

### 3.4. Subgroup & sensitivity analysis

Subgroup analyses compared RCTs and non-RCTs.At 4 weeks, TBUT improved in both RCTs (SMD = 1.35, 95% CI: [0.67, 2.03]) and non-RCTs (SMD = 1.09, 95% CI: [0.34, 1.85]); the between-group difference was not statistically significant (*P* = .51). For the Schirmer *I* test, RCTs reported SMD = 0.31 and non-RCTs reported SMD = −0.15 (*P* = .078). TBUT was also evaluated across follow-up durations of 2, 4, and 12 weeks, with no significant difference among time points (*P* = .847). Sensitivity analysis using Hartung-Knapp-adjusted random-effects models showed stable results, and no single study significantly altered the overall estimates.

### 3.5. Compliance and safety

The compliance rate across the included studies was high, with an overall adherence of 96.8%. This suggests that patients were generally well-motivated and followed the prescribed treatment regimen consistently. In terms of safety, the treatment with 2% rebamipide was well-tolerated among participants. The most commonly reported adverse events were mild, including dysgeusia (altered taste) and nasopharyngitis (inflammation of the nasal passages). Importantly, no major ocular adverse events were reported during the treatment period. These findings indicate that 2% rebamipide ophthalmic suspension is both effective and safe for long-term use in the management of DED, with minimal safety concerns.

### 3.6. Publication bias

**Figure 4.**
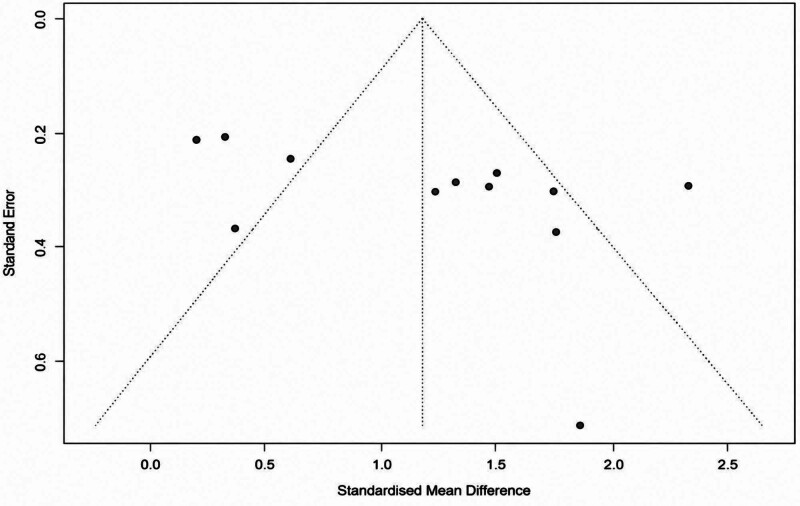
Funnel Plot for TBUT at 4 weeks follow-up. TBUT = tear breakup time.

Funnel plot analysis of TBUT at 4 weeks (12 studies, excluding Y. Eom) suggested slight visual asymmetry. However, Egger’s regression test for funnel plot asymmetry did not show statistically significant publication bias (*t* = 2.20, df = 10, intercept = 5..36 (SE = 2.4303), 95% CI: 0.59–10.12, *P* = .052), as shown in Figure 4. Although close to the conventional significance threshold, this result suggests that any small-study effects are unlikely to have substantially impacted the pooled estimates.

### 3.7. Quality assessment and risk of bias

A total of 6 RCTs were included. All studies were deemed to be of high-quality and showed a low risk of bias, except for some concerns in the overall bias for Jin et al due to the absence of blinding, which introduces potential bias in the randomization process. The quality of the non-randomized studies was evaluated using the National Institutes of Health Quality Assessment Tool for Before-After (Pre-Post) Studies With No Control Group and the Joanna Briggs Institute Critical Appraisal Checklist for Case Series. All studies included in the analysis showed a fair to good quality rating. Risk of bias assessment is detailed in Figure S1, Supplemental Digital Content, https://links.lww.com/MD/R718, and Tables S1(A–C), Supplemental Digital Content, https://links.lww.com/MD/R717, and Tables S2(A–D), Supplemental Digital Content, https://links.lww.com/MD/R717.

## 4. Discussion

This meta-analysis demonstrated that 2% rebamipide ophthalmic suspension significantly improves tear film stability in patients with DED. The pooled data revealed a large increase in tear breakup time (TBUT) (SMD = 1.12; 95% CI, 0.79–1.45; *P* < .0001), indicating a robust therapeutic effect. This degree of change exceeds the minimum clinically important difference of 0.5–1.0 seconds reported in prior studies, suggesting meaningful improvement in tear film stability. In contrast, Schirmer *I* test values showed minimal and statistically nonsignificant improvement (SMD = 0.04; *P* = .83), reinforcing that rebamipide primarily targets the mucin-layer rather than aqueous tear production.

Fluorescein ocular surface staining scores (SMD = −0.68; *P* = .051) and patient-reported outcomes (SMD = −1.17; *P* = .055) showed trends toward improvement, though these results did not reach statistical significance and should be interpreted cautiously. These effects are biologically plausible, given rebamipide’s ability to promote goblet cell proliferation and mucin secretion. The treatment exhibited a favorable safety profile, with adherence rates of 96.8% and only mild adverse events, most commonly dysgeusia and nasopharyngitis.

Our findings are consistent with previous trials, such as Kinoshita et al^[42]^ who reported a 1.5-second TBUT improvement over 52 weeks, and Ballesteros-Sánchez et al^[43]^ who found a comparable pooled effect size (SMD = 1.08). Eom et al^[44]^ however, observed Schirmer improvement: likely due to a higher proportion of aqueous-deficient DED patients, underscoring the importance of subtype-specific efficacy. Histological work by Kase et al^[45]^ further corroborates our results, demonstrating goblet cell proliferation following rebamipide treatment, consistent with mucin-driven mechanisms.

When compared with diquafosol, prior work by Takahashi et al indicated non-inferior TBUT benefits and superior symptom relief with rebamipide. Our trend-level DEQS findings,^[46]^ while not statistically significant, are directionally consistent with these results. Additionally, other randomized trials have shown comparable efficacy between rebamipide and diquafosol in improving TBUT, corneal/conjunctival staining, and symptom scores, with only rebamipide demonstrating statistically significant gains in Schirmer test values.^[47]^ Another comparative trial in office workers reported equivalent TBUT and DEQS improvements, though diquafosol was preferred by some patients for comfort.^[48]^ Compared to cyclosporine A (CsA), rebamipide has shown superior improvements in TBUT and OSDI scores in a recent Indian RCT.^[49]^ These differences likely stem from rebamipide’s rapid mucin-enhancing action versus CsA’s slower immunomodulatory mechanism. Overall, these findings support rebamipide’s comparable, and in some contexts superior, efficacy to established agents such as diquafosol and CsA, with an excellent safety profile and rapid onset of action.

Subgroup analysis in our study revealed more pronounced efficacy in Asian populations, with 10 of 13 included studies originating in Japan or Korea.^[29–39]^ While this may reflect geographic research clustering, ethnic or genetic differences in tear film composition or mucin gene expression cannot be ruled out and warrant further investigation. Epidemiological data suggest that East Asian patients more frequently exhibit the “short TBUT” or mucin-deficient subtype of DED,^[50]^ which may respond more favorably to mucin secretagogues such as rebamipide. Genetic studies have identified distinct MUC5AC haplotypes under positive selection in East Asians that are largely absent in European populations, potentially influencing baseline mucin production.^[51]^ Furthermore, polymorphisms in cytokine genes such as IL-6, which is involved in ocular surface inflammation, also vary by ethnicity and may affect therapeutic response.^[52]^ These population-level pharmacogenetic differences highlight the need for cross-ethnic validation and may inform personalized DED management strategies in the future.

Moderate to high heterogeneity (*I*^2^ = 83.6%) was noted across studies. Subgroup analyses based on treatment duration, DED subtype, and control type did not fully explain this variability. Nonetheless, sensitivity analyses using Hartung-Knapp adjustments confirmed the robustness of primary estimates. Egger’s test approached statistical significance (*P* = .052), suggesting possible small-study effects or publication bias. However, no major asymmetry was observed in the funnel plot, and inclusion of non-RCTs did not significantly influence pooled estimates (*P* = .51 for TBUT), supporting the validity of findings.

This review offers several strengths. It is the first meta-analysis to assess rebamipide’s effect on optical quality outcomes, including higher-order aberrations, and its efficacy in contact lens-associated dry eye. Rigorous methodology was employed, including dual reviewer screening, PRISMA 2020 compliance, and comprehensive sensitivity testing.

### 4.1. limitations

Clinical heterogeneity was substantial, reflecting the inclusion of diverse dry eye etiologies such as postoperative and contact lens-related cases, as well as variability in outcome measurement protocols, particularly for FSS. While several studies employed validated grading systems such as the NEI or van Bijsterveld scales, inconsistent use and reporting limited comparability and may have contributed to the borderline significance observed in staining outcomes. Standardization of fluorescein staining assessments in future trials is essential to improve the reliability and interpretability of pooled evidence.

Approximately half of the included studies were non-randomized^[29–31,34,36–38]^ and lacked blinding; however, subgroup analysis showed no significant differences in effect sizes between randomized and non-randomized designs (*P* = .51 for TBUT). The predominance of Asian studies (12 out of 13) limits the generalizability of findings to broader populations, particularly Western cohorts with differing baseline characteristics. Funnel plot asymmetry approached statistical significance (*P* = .052), suggesting possible publication bias, although sensitivity analyses and the absence of a significant Egger test mitigate major concerns.

### 4.2. Clinical implications

This meta-analysis supports the use of 2% rebamipide ophthalmic suspension as a first-line agent for mucin-deficient dry eye, given its rapid onset, favorable safety profile, and mucin-stimulating mechanism. However, its efficacy may be limited in severe aqueous-deficient cases, where adjunctive therapies such as autologous serum or punctal occlusion are often required.^[53,54]^

Compared to agents like cyclosporine or lifitegrast, rebamipide demonstrates a faster therapeutic response and lower cost in many regions, especially Asia.^[15]^ Despite its advantages, most evidence is derived from Asian populations. Future research should prioritize large, well-designed RCTs in Western and multiethnic cohorts to confirm generalizability.

Standardization of outcome measures, particularly fluorescein staining protocols, is essential to reduce heterogeneity across trials. Longer-term studies exceeding 12 weeks are also needed to assess the durability of the effect.

Combination regimens represent a promising direction. Preliminary studies have evaluated rebamipide plus cyclosporine with mixed results,^[55]^ while case reports suggest that combinations such as rebamipide with diquafosol or autologous serum may yield synergistic benefits.^[56]^ Future trials should assess such combinations using factorial or parallel designs targeting multiple tear film layers.

## 5. Conclusions

This meta-analysis demonstrates that rebamipide 2% ophthalmic suspension significantly improves tear film stability, with supportive trends in ocular surface staining and patient-reported symptoms. These effects, mediated through its mucin-promoting mechanism, position rebamipide as a valuable therapeutic option for mucin-deficient and tear instability–dominant dry eye subtypes. Its favorable safety profile, relatively rapid onset of action, and potential cost advantages further support its clinical utility.

However, its limited impact on aqueous production suggests that rebamipide is most effective as monotherapy in mucin-layer deficiency or as part of combination therapy with aqueous-enhancing or anti-inflammatory agents for broader DED phenotypes. Given the geographic concentration of available evidence in Asian populations, future multicenter randomized controlled trials in more diverse cohorts are essential.

Importantly, the standardization of outcome measurement protocols, particularly for fluorescein staining and the inclusion of longer follow-up durations, will be critical for improving evidence consistency and confirming the long-term effectiveness and generalizability of rebamipide in DED management.

## Author contributions

**Data curation:** Meer Murtaza, Asia Batool.

**Conceptualization:** Meer Murtaza, Umar Farooque.

**Supervision:** Tirath Patel.

**Writing – original draft:** Meer Murtaza, Umar Farooque, Asia Batool, Maria Rasool, Nazeer Ahmed, Syeda Hafsa Qadri, Makhzan Ali Akbar, Shahroz Ali, Tirath Patel.

**Writing – review & editing:** Tirath Patel.

## Supplementary Material
