# Supplementary material for: Efficacy of rebamipide for the treatment of dry eye disease: An updated meta-analysis of randomized and non-randomized controlled trials
Source: Medicine (Baltimore). 2026 May 1;105(18):e48424. doi: 10.1097/MD.0000000000048424 (PMC13138440; doi:10.1097/MD.0000000000048424)
Supplement: Supplementary file 1 [file medi-105-e48424-s001.pdf]

Intention-to-treat	Unique ID	Study ID	Experimental	Comparator	Outcome	Weight	D1	D2	D3	D4	D5	Overall
	1	Igarashi et al.	NA	NA	NA	1	+	+	+	+	+	+
	2	Kobashi et al.	NA	NA	NA	1	+	+	+	+	+	+
	3	Teshigawara et al. (	NA	NA	NA	1	+	+	+	+	+	+
	4	Eom et al.	NA	NA	NA	1	+	+	+	+	+	+
	5	Jin et al.	NA	NA	NA	1	!	!	+	+	+	!
	6	Lee et al.	NA	NA	NA	1	+	+	+	+	+	+

+ Low risk
! Some concerns
- High risk

D1 Randomisation process
D2 Deviations from the intended interventions
D3 Missing outcome data
D4 Measurement of the outcome
D5 Selection of the reported result

Supplementary Figure S1**:** Cochrane risk-of-bias (RoB) 2.0 assessment for randomized-controlled trials
