# Supplementary material for: Efficacy of rebamipide for the treatment of dry eye disease: An updated meta-analysis of randomized and non-randomized controlled trials
Source: Medicine (Baltimore). 2026 May 1;105(18):e48424. doi: 10.1097/MD.0000000000048424 (PMC13138440; doi:10.1097/MD.0000000000048424)
Supplement: Supplementary file 2 [file medi-105-e48424-s002.pdf]

	Yes	No	Unclear	Not applicable
• Were there clear criteria for inclusion in the case series?	☑	☐	☐	☐
• Was the condition measured in a standard, reliable way for all participants included in the case series?	☑	☐	☐	☐
• Were valid methods used for identification of the condition for all participants included in the case series?	☑	☐	☐	☐
• Did the case series have consecutive inclusion of participants?	☑	☐	☐	☐
• Did the case series have complete inclusion of participants?	☐	☐	☑	☐
• Was there clear reporting of the demographics of the participants in the study?	☑	☐	☐	☐
• Was there clear reporting of clinical information of the participants?	☑	☐	☐	☐
• Were the outcomes or follow up results of cases clearly reported?	☑	☐	☐	☐
• Was there clear reporting of the presenting site(s)/clinic(s) demographic information?	☐	☑	☐	☐
• Was statistical analysis appropriate?	☑	☐	☐	☐
Overall appraisal: Include ☑ Exclude ☐ Seek further info ☐				

Supplementary Table S1(a): Joanna Briggs Institute (JBI) Critical Appraisal Checklist for Case Series for Koh et al. [29]

	Yes	No	Unclear	Not applicable
• Were there clear criteria for inclusion in the case series?	☑	☐	☐	☐
• Was the condition measured in a standard, reliable way for all participants included in the case series?	☑	☐	☐	☐
• Were valid methods used for identification of the condition for all participants included in the case series?	☑	☐	☐	☐
• Did the case series have consecutive inclusion of participants?	☐	☐	☑	☐
• Did the case series have complete inclusion of participants?	☐	☐	☑	☐
• Was there clear reporting of the demographics of the participants in the study?	☐	☑	☐	☐
• Was there clear reporting of clinical information of the participants?	☑	☐	☐	☐
• Were the outcomes or follow up results of cases clearly reported?	☑	☐	☐	☐
• Was there clear reporting of the presenting site(s)/clinic(s) demographic information?	☐	☐	☑	☐
• Was statistical analysis appropriate?	☑	☐	☐	☐

Overall appraisal:	Include ☑	Exclude ☐	Seek further info ☐

Supplementary Table S1(b): JBI Critical Appraisal Checklist for Case Series for Ueda et al. [30]

	Yes	No	Unclear	Not applicable
• Were there clear criteria for inclusion in the case series?	☑	☐	☐	☐
• Was the condition measured in a standard, reliable way for all participants included in the case series?	☑	☐	☐	☐
• Were valid methods used for identification of the condition for all participants included in the case series?	☑	☐	☐	☐
• Did the case series have consecutive inclusion of participants?	☐	☐	☑	☐
• Did the case series have complete inclusion of participants?	☐	☐	☑	☐
• Was there clear reporting of the demographics of the participants in the study?	☑	☐	☐	☐
• Was there clear reporting of clinical information of the participants?	☑	☐	☐	☐
• Were the outcomes or follow up results of cases clearly reported?	☑	☐	☐	☐
• Was there clear reporting of the presenting site(s)/clinic(s) demographic information?	☐	☑	☐	☐

• Was statistical analysis appropriate?	☑	☐	☐	☐
Overall appraisal: Include ☑ Exclude ☐ Seek further info ☐				

Supplementary Table S1(c): JBI Critical Appraisal Checklist for Case Series for Sakane et al. [37]

Criteria	Yes	No	Other (CD, NR, NA)*
1. Was the study question or objective clearly stated?	✓		
2. Were eligibility/selection criteria for the study population prespecified and clearly described?	✓		
3. Were the participants in the study representative of those who would be eligible for the test/service/intervention in the general or clinical population of interest?	✓		
4. Were all eligible participants that met the prespecified entry criteria enrolled?			✓
5. Was the sample size sufficiently large to provide confidence in the findings?	✓		
6. Was the test/service/intervention clearly described and delivered consistently across the study population?	✓		
7. Were the outcome measures prespecified, clearly defined, valid, reliable, and assessed consistently across all study participants?	✓		
8. Were the people assessing the outcomes blinded to the participants' exposures/interventions?			✓
9. Was the loss to follow-up after baseline 20% or less? Were those lost to follow-up accounted for in the analysis?			✓
10. Did the statistical methods examine changes in outcome measures from before to after the intervention? Were statistical tests done that provided p values for the pre-to-post changes?	✓		
11. Were outcome measures of interest taken multiple times before the intervention and multiple times after the intervention (i.e., did they use an interrupted time-series design)?			✓
12. If the intervention was conducted at a group level (e.g., a whole hospital, a community, etc.) did the statistical analysis take into account the use of individual-level data to determine effects at the group level?			✓
**Quality Rating (Good, Fair, or Poor): Good**<br><br>*CD, cannot determine; NA, not applicable; NR, not reported			

Supplementary Table S2(a): National Institutes of Health (NIH) Quality Assessment Tool for Before-After (Pre-Post) Studies With No Control Group for Igarashi T et al. [31]

Criteria	Yes	No	Other (CD, NR, NA)*
1. Was the study question or objective clearly stated?	✓		
2. Were eligibility/selection criteria for the study population prespecified and clearly described?	✓		
3. Were the participants in the study representative of those who would be eligible for the test/service/intervention in the general or clinical population of interest?		✓	
4. Were all eligible participants that met the prespecified entry criteria enrolled?			✓
5. Was the sample size sufficiently large to provide confidence in the findings?	✓		
6. Was the test/service/intervention clearly described and delivered consistently across the study population?	✓		
7. Were the outcome measures prespecified, clearly defined, valid, reliable, and assessed consistently across all study participants?	✓		
8. Were the people assessing the outcomes blinded to the participants' exposures/interventions?			✓
9. Was the loss to follow-up after baseline 20% or less? Were those lost to follow-up accounted for in the analysis?	✓		
10. Did the statistical methods examine changes in outcome measures from before to after the intervention? Were statistical tests done that provided p values for the pre-to-post changes?	✓		
11. Were outcome measures of interest taken multiple times before the intervention and multiple times after the intervention (i.e., did they use an interrupted time-series design)?			✓
12. If the intervention was conducted at a group level (e.g., a whole hospital, a community, etc.) did the statistical analysis take into account the use of individual-level data to determine effects at the group level?			✓
**Quality Rating (Good, Fair, or Poor): Fair**<br><br>*CD, cannot determine; NA, not applicable; NR, not reported			

Supplementary Table S2(b): NIH Quality Assessment Tool for Before-After (Pre-Post) Studies With No Control Group for Igarashi T et al. [34]

Criteria	Yes	No	Other (CD, NR, NA)*
1. Was the study question or objective clearly stated?	✓		
2. Were eligibility/selection criteria for the study population prespecified and clearly described?	✓		
3. Were the participants in the study representative of those who would be eligible for the test/service/intervention in the general or clinical population of interest?	✓		
4. Were all eligible participants that met the prespecified entry criteria enrolled?			✓
5. Was the sample size sufficiently large to provide confidence in the findings?	✓		
6. Was the test/service/intervention clearly described and delivered consistently across the study population?	✓		
7. Were the outcome measures prespecified, clearly defined, valid, reliable, and assessed consistently across all study participants?	✓		
8. Were the people assessing the outcomes blinded to the participants' exposures/interventions?			✓
9. Was the loss to follow-up after baseline 20% or less? Were those lost to follow-up accounted for in the analysis?			✓
10. Did the statistical methods examine changes in outcome measures from before to after the intervention? Were statistical tests done that provided p values for the pre-to-post changes?	✓		
11. Were outcome measures of interest taken multiple times before the intervention and multiple times after the intervention (i.e., did they use an interrupted time-series design)?			✓
12. If the intervention was conducted at a group level (e.g., a whole hospital, a community, etc.) did the statistical analysis take into account the use of individual-level data to determine effects at the group level?			✓
**Quality Rating (Good, Fair, or Poor): Good**<br><br>*CD, cannot determine; NA, not applicable; NR, not reported			

Supplementary Table S2(c): NIH Quality Assessment Tool for Before-After (Pre-Post) Studies With No Control Group for Teshigawara et al. [36]

Criteria	Yes	No	Other (CD, NR, NA)*
1. Was the study question or objective clearly stated?	✓		
2. Were eligibility/selection criteria for the study population prespecified and clearly described?	✓		
3. Were the participants in the study representative of those who would be eligible for the test/service/intervention in the general or clinical population of interest?	✓		
4. Were all eligible participants that met the prespecified entry criteria enrolled?	✓		
5. Was the sample size sufficiently large to provide confidence in the findings?	✓		
6. Was the test/service/intervention clearly described and delivered consistently across the study population?	✓		
7. Were the outcome measures prespecified, clearly defined, valid, reliable, and assessed consistently across all study participants?	✓		
8. Were the people assessing the outcomes blinded to the participants' exposures/interventions?			✓
9. Was the loss to follow-up after baseline 20% or less? Were those lost to follow-up accounted for in the analysis?			✓
10. Did the statistical methods examine changes in outcome measures from before to after the intervention? Were statistical tests done that provided p values for the pre-to-post changes?	✓		
11. Were outcome measures of interest taken multiple times before the intervention and multiple times after the intervention (i.e., did they use an interrupted time-series design)?			✓
12. If the intervention was conducted at a group level (e.g., a whole hospital, a community, etc.) did the statistical analysis take into account the use of individual-level data to determine effects at the group level?			✓
**Quality Rating (Good, Fair, or Poor): Good**<br><br>*CD, cannot determine; NA, not applicable; NR, not reported			

Supplementary Table S2(d): NIH Quality Assessment Tool for Before-After (Pre-Post) Studies with No Control Group for Teshigawara et al. [38]
